# Early laparoscopic cholecystectomy in severely comorbid patients with acute cholecystitis: results of a monocentric study

**DOI:** 10.2144/fsoa-2023-0185

**Published:** 2024-05-14

**Authors:** Mohamed S Jarrar, Malek Barka, Mehdi Chahed, Radhouane Toumi, Ameni Beizig, Mohamed H Mraidha, Fehmi Hamila, Sabri Youssef

**Affiliations:** 1 Department of General & Digestive Surgery – Farhat Hached University Hospital – Sousse/Faculty of Medicine of Sousse, 4000, Tunisia; 2 Intensive Care Unit – Farhat Hached University Hospital – Sousse/Faculty of Medicine of Sousse, 4000, Tunisia; 3 Emergency Department – Regional Hospital of Kasserine/Faculty of Medicine of Sousse, 4000, Tunisia

**Keywords:** acute cholecystitis, American Society of Anesthesiologists (ASA) score, early laparoscopic cholecystectomy, high-risk patients, outcomes

## Abstract

**Aim:** The aim is to evaluate laparoscopic cholecystectomy safety based on American Society of Anesthesiologists score for acute cholecystitis in patients with comorbidities. **Patients & methods:** This is retrospective study of patients who underwent laparoscopic cholecystectomy for acute cholecystitis between 2003 and 2021. According to their respective ASA-score, patients were divided into group 1: ASA1-2 and group 2: ASA3-4. **Results:** We collected 578 patients. Even though the gangrenous forms were more frequent and the operative time was longer in group 2, laparoscopic cholecystectomy seems safe and effective. We didn't observe any differences in terms of intraoperative incidents, open conversion rate, or postoperative complications compared with other patients. **Conclusion:** ASA3-4 patients with acute cholecystitis don't face elevated risks of complications or mortality during laparoscopic cholecystectomy.

## Plain language summary

This study, involving 578 patients with acute cholecystitis, assessed the safety of early laparoscopic cholecystectomy based on their health scores. Despite longer operative times and more gangrenous forms in higher-scored patients, laparoscopic cholecystectomy was found to be safe and effective. No significant differences in complications or mortality were observed compared with lower-scored patients. In conclusion, early laparoscopic cholecystectomy is considered a safe option for patients with higher health scores facing acute cholecystitis.

## Tweetable abstract

Study assessed laparoscopic cholecystectomy safety in high-risk patients with acute cholecystitis based on ASA scores. Despite longer operative times, it's a safe and effective option. #CholecystectomySafety

## Abstract (plain language summary)

Laparoscopic cholecystectomy is established as the gold standard for treating acute lithiasic cholecystitis (ALC), as endorsed by guidelines recommending its immediate use to mitigate potential complications.

The investigation revealed that, notwithstanding extended surgical durations and a heightened occurrence of gangrenous ALC among high-risk patients, early laparoscopic cholecystectomy remained a secure and efficacious intervention.

Significant disparities in intraoperative occurrences, conversions to open surgery, or postoperative complications were not observed between the two patient groups.

Nonetheless, patients at elevated risk encountered prolonged postoperative hospitalizations. High-risk individuals exhibited a higher prevalence of comorbidities like diabetes and cardiovascular ailments; however, these factors did not substantially affect surgical outcomes.

While systemic inflammation and specific comorbidities did influence outcomes in the high-risk cohort, laparoscopic cholecystectomy continued to deliver benefits in the form of reduced postoperative complications and abbreviated hospital stays.

## Graphical abstract

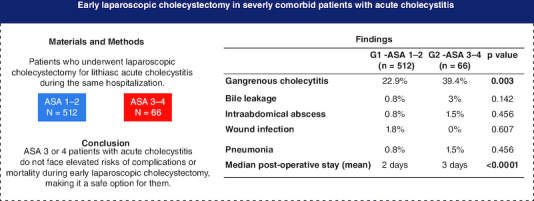

Cholecystectomy stands as the definitive treatment for acute lithiasic cholecystitis (ALC) and complications stemming from cholelithiasis. Laparoscopic cholecystectomy (LC) has proven its safety and effectiveness in addressing ALC [1,2]. Neglecting to provide definitive treatment after an initial ALC episode leads to additional complications associated with cholelithiasis, affecting 20–30% of patients [3]. Current guidelines advocate for LC during the same hospitalization, as it carries a lower risk of conversion to laparotomy and postoperative complications compared with delayed cholecystectomy [^4-7^].

However, managing ALC in elderly patients with comorbidities presents a significant challenge. This study aims to assess the safety of LC for ALC in patients categorized based on their ASA (American Society of Anesthesiologists) score [8].

## Materials & methods

This retrospective observational study included patients who underwent early LC for ALC between January 2003 and December 2021. ALC diagnosis followed the Tokyo Guidelines (TG) criteria [9], and patients were tracked for a minimum of 30 postoperative days.

Patients diagnosed with ALC received empiric antibiotic therapy following guidelines [10]. LC was performed using a standard four-trocar technique, with a right sub-costal incision for open surgery conversion if necessary. All removed gallbladder specimens were histopathologically confirmed as ALC. Patients were followed up for at least 30 days post-discharge. Exclusion criteria encompassed chronic cholecystitis, calculous cholecystitis, and other concurrent acute biliary conditions (cholangitis, choledocholithiasis, or acute pancreatitis). The study ensured patient confidentiality and anonymity and received a waiver from the Institutional Research and Ethics Committee.

A total of 578 patients underwent early LC and were categorized into two groups: Group 1 (G1) with ASA scores of 1 or 2, and Group 2 (G2) with scores of 3 or 4.

Data collected included patient demographics, co-morbidities, previous abdominal surgeries, laboratory and radiological findings, operative details, and postoperative outcomes. Severity grades were assigned following TG guidelines [9]. There were no missing data.

Data analysis used Epi-info software (version 8). Descriptive results were presented as counts and proportions for categorical variables, while continuous variables (not normally distributed) were represented as median with interquartile range (IQR). Statistical analysis employed the χ^2^ test or the exact Fisher test for categorical variables and the Mann-Whitney U test for continuous variables, with significance at p < 0.05. This study adhered to STROBE guidelines for observational studies [11].

## Results

The study included 578 patients: 512 (88.6%) in G1 (ASA1: 302, ASA2: 210) and 66 (11.4%) in G2 (ASA3: 65, ASA4: 1). All underwent early LC for mild (180) or moderate (398) LAC.

Table 1 displays baseline characteristics. Median ages were 53 in G1 and 70.5 in G2. Gender distribution showed no significant difference between groups, with 68.8% females in G1 and 63.6% in G2 (p = 0.401). G2 had higher rates of diabetes (53% vs 13.5%) and cardiovascular pathologies, particularly hypertension (80.3% vs 21.9%) and ischemic heart disease (31.8% vs 2.1%). Laboratory markers indicated hyperleukocytosis in both groups, with notable differences in gamma-glutamyl transpeptidase (81 UI/l in G2 vs 37 UI/l in G1; p = 0.013) and aspartate aminotransferase (27.5 UI/l in G2 vs 23 UI/l in G1; p = 0.015). Radiological findings showed no significant differences, and ALC severity was comparable between groups according to TG criteria.

**Table 1.** Patient characteristics.

	Total (n = 578)	G1 – ASA 1–2 (n = 512)	G2 – ASA 3–4 (n = 66)	p-value
**Median age, years (range)**	55 (39–75)	53 (39–62)	70.5 (62.5–75)	**<0.0001**
Female sex, n (%)	394 (68.2)	352 (68.8)	42 (63.6)	0.401
Comorbidity, n (%)				
Diabetes mellitus	104 (18)	69 (13.5)	35 (53)	**<0.0001**
Hypertension	165 (28.5)	112 (21.9)	53 (80.3)	**<0.0001**
Ischemic heart disease	32 (5.5)	11 (2.1)	21 (31.8)	**<0.0001**
Respiratory insufficiency	14 (2.4)	12 (2.3)	2 (3)	0.668
Renal insufficiency	1 (0.2)	0 (0)	1 (1.5)	0.114
Previous abdominal surgery, n (%)	54 (9.3)	46 (9)	8 (12.1)	0.410
Median symptom duration, days (IQR)^†^	4 (3–7)	4 (3–6)	4 (3–7)	0.238
Median laboratory results, unit (IQR)				
WBC, n × 10^9^	11.9 (9–15.9)	11.9 (9.1–14.65)	12.1 (8.45–15.9)	0.909
Total bilirubin, micromol/l	14 (10–20)	14 (10–19)	15 (10–20)	0.461
Alkaline phosphatase, UI/l	95 (63–191)	95 (62.5–140.5)	99 (68–191)	0.294
Gamma glutamyl tanspeptidase, UI/l	39.5 (21–142)	37 (21–70)	81 (27–142)	**0.013**
Aspartate aminotransferase, UI/l	23 (17–74.25)	23 (17–36)	27.5 (19–74.25)	**0.015**
Alanine aminotansferase, UI/l	23 (16–49)	23 (16–37)	26.5 (16.25–49)	0.309
Radiological findings, n (%)				
Gallbladder wall thickening	516 (89.3)	455 (88.9)	61 (92.4)	0.379
Striated wall	159 (27.5)	135 (26.4)	24 (36.4)	0.087
Pericholecystic fluid	58 (10)	47 (9.2)	11 (16.7)	0.057
Impacted stones in the gallbladder neck	209 (36.2)	192 (37.5)	17 (25.8)	0.062
Severity grade, n (%)				0.316
Mild (grade I)	180 (31.1)	163 (31.8)	17 (25.8)	
Moderate (grade II)	398 (68.9)	349 (68.2)	49 (74.2)	

Terms in bold indicate parameters where the difference is significant.

† Median symptom duration is the time between the start of symptoms and surgery.

ASA: American society of anesthesiologists; G: Group; IQR: Interquartile range; WBC: White blood count.

Table 2 summarizes operative findings, revealing a longer operative time in G2 (median 90 min vs 80 min in G1; p = 0.015) and a higher rate of gangrenous ALC (39.4% vs 22.9% in G1; p = 0.003). Conversion to open surgery (G1 13.1% vs G2 21.2%; p = 0.073) and intra-operative complications did not significantly differ between groups.

**Table 2.** Operative parameters.

	Total (n = 578)	G1 – ASA 1–2 (n = 512)	G2 – ASA 3–4 (n = 66)	p-value
Median operative time, min (IQR)	80 (60–120)	80 (60–100)	90 (70–120)	**0.015**
Open conversion, n (%)	81 (14)	67 (13.1)	14 (21.2)	0.073
Intra-operative cholangiography, n (%)	13 (2.3)	12 (2.3)	1 (1.5)	1
Gangrenous cholecystitis, n (%)	143 (24.7)	117 (22.9)	26 (39.4)	**0.003**
Intra-operative morbidity, n (%)				
Bleeding >500 ml	15 (2.6)	12 (2.3)	3 (4.5)	0.398
Bile duct injury	1 (0.2)	0 (0)	1 (1.5)	0.114
Bowel injury	1 (0.2)	1 (0.2)	0 (0)	1
Intra-operative mortality, n (%)	0 (0)	0 (0)	0 (0)	–

Terms in bold indicate parameters where the difference is significant.

ASA: American society of anesthesiologists; G: Group; IQR: Interquartile range.

Table 3 presents postoperative outcomes, showing no significant differences in major complications, bile leakage, respiratory, renal complications, wound infection, or intra-abdominal abscesses. No reoperations or deaths occurred. However, postoperative hospital stay was significantly longer in G2 (median 3 days vs 2 days in G1, p < 0.05).

**Table 3.** Postoperative outcomes.

	Total (n = 578)	G1 – ASA 1–2 (n = 512)	G2 – ASA 3–4 (n = 66)	p-value
Bile leakage, n (%)	6 (1)	4 (0.8)	2 (3)	0.142
Intraabdominal abscess, n (%)	5 (0.9)	4 (0.8)	1 (1.5)	0.456
Wound infection, n (%)	9 (1.6)	9 (1.8)	0 (0)	0.607
Pneumonia, n (%)	5 (0.9)	4 (0.8)	1 (1.5)	0.456
Median post-operative stay, days (IQR)	2 (1–3)	2 (1–3)	3 (2–5)	**<0.0001**

ASA: American Society of Anesthesiologists; G: Group; IQR: Interquartile range.

## Discussion

Our results indicate that despite higher rates of gangrenous ALC and longer operative times in high-risk patients, LC remains safe and effective. We observed no significant differences in intraoperative incidents, conversion rates, or postoperative complications. However, high-risk patients experienced longer postoperative stays. The results of the present study are quite generalizable seen as they reflect the real-life cohort of all consecutive patients admitted to a third level hospital, during 19 years.

Initiating our study, group 1 had 512 patients, and group 2 had 66. Despite a notable difference, it's crucial to note that this dissimilarity doesn't undermine our findings' validity. Scientific inquiry relies on precise methodologies and valid results, and adept statistical analyses can accommodate varied sample sizes, ensuring the credibility of conclusions. Research quality hinges on methodological rigor rather than specific group magnitudes.

Patients with comorbidities, linked to age-related changes, are more vulnerable to severe infections like gangrenous cholecystitis due to compromised immune function. Concurrent diseases heighten the risk of complications, including gallbladder gangrene, while diminished tissue regeneration poses challenges in recovery. Heightened pain threshold and poorly localized sensations contribute to delayed diagnosis. Comorbid patients, often managing multiple medications, may misattribute symptoms. Treatments like analgesics can mask underlying issues, leading to potential misguidance. Delays in consultation can exacerbate cholecystitis, fostering the development of gangrenous forms.

Comorbidities can have a significant influence on operative difficulties and postoperative complications in patients. Comorbidities such as diabetes, hypertension, cardiovascular or respiratory diseases, among others, can increase the risks associated with surgery. These pre-existing conditions can make the procedure more complex due to the fragility or resistance of the tissues, the effect on the immune system or the reaction to the drugs used during the operation. Additionally, patients with comorbidities may be more vulnerable to postoperative complications, such as infections, wound healing issues, bleeding disorders, or general recovery issues. Therefore, it is essential that the medical team take these factors into account when planning the procedure, adapting surgical protocols and providing careful postoperative care to minimize risks and ensure the best possible recovery for patients with comorbidities [12].

With the continuous increase in the proportion of blemished elderly in the global population, this patient group tends to undergo elective and emergency surgeries frequently, including biliary surgery, which appears to have more postoperative complications [13,14]. Recent advances in laparoscopic surgery have expanded the indications for surgical treatment of biliary pathology, including ALC, even in high risk patients [^15-18^]. This fact has been confirmed by several studies, showing the superiority of ELC in terms of cost-effectiveness and quality of life of patients, without an increase in the morbidity rate compared with delayed laparoscopic cholecystectomy [^4-7^]. The benefits of one-stage management during the initial index admission have been reported by some randomized trials, demonstrating that urgent surgery was associated with a significant reduction in the total hospital staywith no significant difference in the conversion rate or complication rate between urgent and interval surgery [18].

Riall looked at the results and costs of cholecystectomies for ALC in 29.818 patients with high operative risk. In this study, the authors concluded that cholecystectomy for ALC in these patients should be performed during the initial hospitalization to prevent recurrent episodes of cholecystitis, multiple readmissions, higher readmission rates, and increased costs [16]. Yi compared three groups of patients with ALC: 33 ASA1, 79 ASA2 and 25 ASA3. He concluded that ELC for ALC may be an effective treatment option in high-risk elderly patients. Indeed, the open conversion rate, diet delay, postoperative complications and hospital stay were similar in the three groups [17].

However, in a study of 725 patients operated on for ALC, Osterman demonstrated that patients with severe systemic disease had an increased risk of complications but not death after emergency surgery. The risk is lower for elective procedures, but a substantial proportion will have new gallstone complications before elective surgery [19]. The degree of systemic inflammation was the main factor influencing the unfavourable outcome of LC in patients at high operative risk. Among comorbidities, diabetes was associated with increased postoperative surgical and systemic morbidity, while stroke and chronic renal failure were correlated with an elevated risk of cardiovascular complications. With adequate perioperative care, those at high operative risk have much to gain from the benefits of a minimally invasive approach, which results in a lower postoperative complication rate and reduced hospital stay [20].

This study, conducted in Central-Eastern Tunisia, represents the first study into acute cholecystitis among patients with severe comorbidities in the Tunisian context. Notable for its extensive sample size, our research establishes a robust and representative database, contributing significantly to medical research. Distinguished by its comprehensive scale, the study enables a thorough analysis of the clinical and epidemiological characteristics of acute cholecystitis within a population facing health challenges in central Tunisia. This study, while robust in its population size, has limitations stemming from its retrospective nature and potential selection bias. The lack of standardization for alternative ALC treatments in the service precludes determining early LC's superiority over nonsurgical options for comorbid patients [21,22]. On the other hand, despite the relative frequency of diabetes, arterial hypertension and myocardial ischemia in our population, we were unable to compare subgroups concerning these conditions due to insufficient data availability. Future comparative and prospective studies are needed to guide optimal care for these patients.

## Conclusion

There is no increased risk of intra and postoperative complications nor open conversion in ASA three or four patients with ALC. There is no increase in mortality either. Therefore, early laparoscopic cholecystectomy could be safely offered for ASA three or four patients with mild or moderate acute cholecystitis.

However, a discussion is needed with patients who are potential candidates for surgery, clearly explaining the risks and benefits. If not an emergency candidate, ASA three to four patients might be prioritized for planned procedures due to the high risk of second gallstone complications, while ASA1 and ASA2 patients are operated on in an emergency situation.
